# The effects of male age on sperm analysis by motile sperm organelle morphology examination (MSOME)

**DOI:** 10.1186/1477-7827-10-19

**Published:** 2012-03-19

**Authors:** Liliane FI Silva, Joao Batista A Oliveira, Claudia G Petersen, Ana L Mauri, Fabiana C Massaro, Mario Cavagna, Ricardo LR Baruffi, José G Franco

**Affiliations:** 1 Department of Gynaecology and Obstetrics, Botucatu Medical School, São Paulo State University - UNESP, Botucatu, Brazil; 2 Center for Human Reproduction Prof Franco Jr, Ribeirao Preto, Brazil; 3 Paulista Centre for Diagnosis, Research and Training, Ribeirao Preto, Brazil; 4 Women's Health Reference Center, Perola Byington Hospital, Sao Paulo, Brazil

**Keywords:** Male age, MSOME, IMSI, Sperm morphology, DNA damage

## Abstract

**Background:**

This study aimed to investigate the influence of age on sperm quality, as analysed by motile sperm organelle morphology examination (MSOME).

**Methods:**

Semen samples were collected from 975 men undergoing evaluation or treatment for infertility. Sperm cells were evaluated at 8400× magnification using an inverted microscope equipped with Nomarski (differential interference contrast) optics. Two forms of spermatozoa were considered: normal spermatozoa and spermatozoa with large nuclear vacuoles (LNV, defined as vacuoles occupying > 50% of the sperm nuclear area). At least 200 spermatozoa per sample were evaluated, and the percentages of normal and LNV spermatozoa were determined. The subjects were divided into three groups according to age: Group I, less than or equal to 35 years; Group II, 36-40 years; and Group III, greater than or equal to 41 years.

**Results:**

There was no difference in the percentages of normal sperm between the two younger (I and II) groups (*P >*0.05). The percentage of normal sperm in the older group (III) was significantly lower than that in the younger (I and II) groups (*P *< 0.05). There was no difference in the percentage of LNV spermatozoa between the younger (I and II) groups (*P >*0.05). The percentage of LNV spermatozoa was significantly higher in the older group (III) than in the younger (I and II) groups (*P *< 0.05). Regression analysis demonstrated a significant decrease in the incidence of normal sperm with increasing age (*P *< 0.05; r = -0.10). However, there was a significant positive correlation between the percentage of spermatozoa with LNV and male age (*P *< 0.05, r = 0.10).

**Conclusion:**

The results demonstrated a consistent decline in semen quality, as reflected by morphological evaluation by MSOME, with increased age. Considering the relationship between nuclear vacuoles and DNA damage, these age-related changes predict that increased paternal age should be associated with unsuccessful or abnormal pregnancy as a consequence of fertilisation with damaged spermatozoa. Given that sperm nuclear vacuoles can be evaluated more precisely at high magnification, these results support the routine use of MSOME for ICSI as a criterion for semen analysis.

## Background

Male fertility is an important contributor to the conception potential of a couple. The evaluation of male fertility is generally based on the examination of sperm parameters and sperm functionality. Furthermore, epidemiological evidence suggests that there is a decline in semen quality (e.g., volume, motility, and morphology) and male fertility associated with increased male age [1-12]. In addition, advanced paternal age has been implicated in an increased frequency of miscarriages [5,13,14], autosomal dominant disorders, aneuploidies, and other diseases [15-18].

Among all of the semen parameters studied, sperm morphology has been the best indicator of male fertility because it reflects on the functional competence of the sperm, although none of the semen parameters, either alone or in combination, can be considered definitive. Diverse studies, originating principally from IVF programmes and intrauterine insemination, corroborate the sensitivity of morphology as a prognostic factor [19]. However, the value of traditional semen analysis has been debated.

Innovative methods for the selection of sperm in assisted reproduction techniques (ARTs) have been published, providing new insights into the correlation between sperm quality and clinical results. To test the hypothesis that subtle sperm organelle malformations are associated with ART results, Bartoov *et al. *[20] proposed a new method for real-time evaluation of sperm morphology that is termed the motile sperm organelle morphology examination (MSOME). MSOME utilises an inverted light microscope equipped with high-power Nomarski optics enhanced by digital imaging to achieve a magnification above > 6000×. This magnification is sufficiently high to evaluate spermatozoa according to their fine nuclear morphology, and it is much higher than the magnification typically used by embryologists to select spermatozoa for ICSI (200x to 400x) or even that employed in routine semen examination (1000x). This method led to the development of intracytoplasmic morphologically selected sperm injection (IMSI), which is based on sperm normality, as defined by MSOME classification, and it is aimed at improving conventional ICSI outcomes by focusing mainly on the correlation between DNA damage and sperm morphological abnormalities that can be observed at high magnification [21-23]. The most important predictor of sperm quality is the extent of impairment of the sperm head by the presence of vacuoles. Vacuoles, which are best observed at high magnification, appear to adversely affect embryo development and seem to be related to abnormal chromatin packaging or to the denaturation and fragmentation of sperm DNA [24-27]. The use of IMSI has revealed that the selection of a morphologically normal sperm nucleus before injection is an important factor in improving fertilisation rates, embryo quality [21,28], the rate of development up to the blastocyst stage [26,29], the rates of implantation and pregnancy after embryo transfer on day 2 or 3 [21,22,28,30-34] or in the blastocyst stage [26], and the likelihood of having a normal healthy child [35]. IMSI also appears to significantly decrease miscarriage rates [21,22,26,32,34].

Although MSOME was developed only as a selection criterion, its application as a method for classifying sperm morphology may improve the evaluation of semen quality with potential clinical repercussions, particularly with regard to ART. The objective of the present study was to better define the value of MSOME by using this technique to investigate the influence of age on sperm quality in a group of men from an infertility clinic.

## Methods

### Population

Semen samples (one per subject) were obtained from 975 men from a random group of couples undergoing infertility investigation and treatment at the Centre for Human Reproduction Prof. Franco Jr.

Written informed consent was obtained from all participants, and this study was approved by the institutional review board of Women's Health Reference Center (Brazil).

### Sample collection

Semen samples were collected in sterile containers by masturbation after a sexual abstinence period of 2-5 days. A portion of each semen sample was immediately processed for MSOME. The liquefied fresh semen samples were prepared using Isolate (Irvine Scientific, USA) discontinuous concentration gradient. The final pellet was resuspended in 0.2 ml of modified human tubal fluid (HTF) medium (Irvine Scientific, Santa Ana, CA, USA) and subsequently sent for MSOME. The remainder of the semen sample was used to analyse standard semen quality parameters according to the World Health Organization guidelines [36] and for sperm DNA fragmentation analysis. DNA fragmentation in spermatozoa was measured using the TdT (terminal deoxyribonucleotidyl transferase)-mediated dUTP nick-end labelling (TUNEL) assay, which was performed using an in situ cell death detection kit with tetramethylrhodamine-labelled dUTP (Roche, Monza, Italy).

### Determination of morphology by MSOME

An aliquot of 1 μl of sperm cell suspension was transferred to a 5 μl microdroplet of modified HTF medium containing 7% polyvinylpyrrolidone (PVP medium; Irvine Scientific). This microdroplet was placed in a sterile glass dish (Fluorodish; World Precision Instruments, USA) under sterile paraffin oil (Ovoil-100; VitroLife, Goteborg, Sweden). The sperm cells, which were suspended in the microdroplet, were placed on a microscope stage above a U Plan Apochromat 100x oil/1.35 objective lens that had previously been covered by a droplet of immersion oil. With this procedure, the suspended motile sperm cells in the observation droplet could be examined at high magnification using an inverted microscope (Eclipse TE 2000 U; Nikon, Japan) equipped with high-power differential interference contrast optics (DIC/Nomarski). The images were captured by a colour video camera that had sufficient resolution to produce high-quality images, which were displayed on a colour video monitor. Morphological evaluation was performed on the monitor screen, and the combined calculated magnification was 8450× (total magnification: objective magnification = 100×; magnification selector = 1.0×; video coupler magnification = 1.0×; calculated video magnification = 84.50).

Two types of spermatozoa observed via MSOME were counted in this study: normal spermatozoa and spermatozoa with large nuclear vacuoles (LNV). A spermatozoon was classified as morphologically normal when it exhibited a normal nucleus as well as a normal acrosome, post-acrosomal lamina, neck and tail and had no cytoplasm around the head [20]. The subcellular organelles were morphologically classified on the basis of the presence of specific malformations; these were defined according to the arbitrary descriptive approach reported by Bartoov *et al. *[20] based on transmission and scanning electron microscopy studies: the acrosome as absent, partial or vesiculated; the post-acrosomal lamina as absent or vesiculated; the neck's abaxial as disordered or showing a cytoplasmic droplet; and the tail as absent, coiled, broken, multi or short.

**Figure 1:**
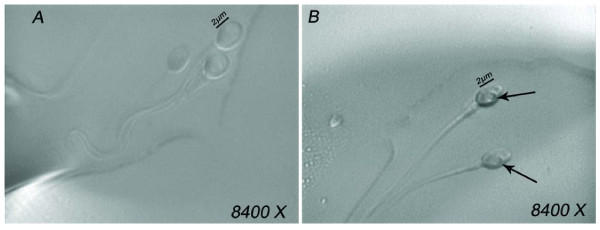
**MSOME**. Human sperm morphology (8450X). A = normal spermatozoa; B = spermatozoa with vacuoles.

The morphological state of the nucleus was defined by its shape and chromatin content, also according to transmission electron microscopy estimations [20,34]. The normal nuclear shape was defined as a smooth, symmetric oval. The normal means for length and width were estimated as 4.75 ± 2.8 and 3.28 ± 0.20 μm [20], respectively, and forms classified as abnormal varied by 2 SD in at least one of the axes (length: ≥ 5.31 or ≤ 4.19 μm, width: > 3.7 or < 2.9 μm). For rapid evaluation of the nuclear shape, a fixed, transparent, celluloid form of sperm nucleus that fit the criteria was superimposed on the examined cell (chablon construction based on ASTM E 1951-2[37]). The criterion for normality of chromatin content was the absence of vacuoles occupying > 4% of the sperm nuclear area. Figure 1A shows normal spermatozoa analysed by MSOME.

LNV spermatozoa were defined according to the Bartoov modified classification, i.e., the presence of one or more vacuoles occupying > 50% of the sperm nuclear area (visual evaluation aided, if necessary, by a celluloid form of a large vacuole superimposed on the examined cell). Figure 1B shows LNV spermatozoa analysed using MSOME.

The same technician performed all sperm selections. As in other sperm morphological analyses, each sperm was evaluated/classified individually in MSOME, and the process was carried out directly on the monitor screen. At least 200 motile spermatozoa per sample were evaluated, and the percentages of normal and LNV spermatozoa were determined. The analysis lasted 30-60 min/sample.

### Quality control

To control for intra-observer variability, multiple fractions of motile spermatozoa were obtained from randomly selected patients. Each sample was observed at least three times by the same observer. A variation of ≈ 0.5% (maximum) was obtained for each parameter analysed: normality of the spermatozoon as a whole, normality of nuclear form, normality of chromatin, spermatozoa with any nuclear vacuoles, and spermatozoa with vacuoles occupying > 50% of the nuclear area. These variations are comparable to those of classical sperm quality parameters [38]. Inter-observer variability was not evaluated because only one observer, blinded to subject identity, performed the entire study.

### Statistical analysis

The data were analysed using the StatsDirect statistical software (Cheshire, UK). The Mann-Whitney U test, Student's *t*-test and chi-squared test were used, as appropriate. Correlations were performed using the Spearman rank correlation test. Patient age and percentages of normal and LNV spermatozoa were treated as continuous variables for regression and correlation analysis. For two-group comparisons, the following ages were used as cut-off points to divide the subjects into groups: Group I: ≤ 35 years, Group II: 36-40 years, and Group III: ≥ 41 years. The level of significance was set at *P *< 0.05.

## Results

Table 1 summarises the general characteristics of the study population. The comparison between the three age groups showed that a significantly higher proportion of older men had fathered at least one child (or a pregnancy that had ended in miscarriage), spontaneously or after fertility treatment, compared with the younger men. Similarly, an increase in the length of the infertile period was also observed with increasing age. Furthermore, as observed in other studies, increased sperm DNA fragmentation was correlated with increasing age. An equal distribution (*P *> 0.05) of the other characteristics was observed for all three groups.

**Table 1:** General characteristics of the three age groups studied

Characteristic	Total	Group I (≤ 35 years)	Group II (36-40 years)	Group III (≥ 41 years)
Patients	975	407	292	276
Age (years)	37.5 ± 6.7	31.7 ± 2.7	37.8 ± 1.3	45.8 ± 5.2
Fathered at least one child	36%	23.1%^a,b^	33.6%^a,c^	57.7%^b,c^
	(351/975)	(94/407)	(98/292)	(159/276)
Duration of infertility (years)	3.7 ± 3.3	3.0 ± 2.2^d,e^	3.6 ± 2.9^d,f^	5.0 ± 4.5^e,f^
Abstinence (mean ± SD)	3.5 ± 1.3	3.6 ± 1.3	3.5 ± 1.4	3.5 ± 1.3
Sperm Parameters* (mean ± SD)				
-volume (ml)	2.7 ± 0.13	2.8 ± 1.2	2.9 ± 1.2	2.6 ± 1.4
-total sperm count x10^6^/ml	61.2 ± 53.8	62.9 ± 53.0	64.8 ± 53.2	56.0 ± 51.8
-motility (rapid+slow progression)%	58.9 ± 17.9	59.9 ± 17.3	57.6 ± 18.7	54.4 ± 19.2
-leukocytes (x10^6^)	0.4 ± 0.9	0.4 ± 0.7	0.4 ± 1.2	0.4 ± 0.7
-vitality (%)	66.5 ± 15.1	68.7 ± 14.1	65.6 ± 15.7	64.2 ± 15.3
Sperm DNA fragmentation (%)	17.1 ± 9.6	15.6 ± 9.1^g,h^	18.1 ± 9.7^g^	18.3 ± 10.2^h^
Varicocele (%)	17	14.7	18.5	18.8
	(166/975)	(60/407)	(54/292)	(52/276)
Tobacco use (%)	11.9	13.8	10.3	10.9
	(116/975)	(56/407)	(30/292)	(30/276)
Regular alcohol use (%)	64.2	65.3	66.4	60.1
	(626/975)	(266/407)	(194/292)	(166/276)
Vitamin supplement use (%)	15.5	15.5	13.7	17.4
	(151/975)	(63/407)	(40/292)	(48/276)

*Categorised according to World Health Organization guidelines [36]

Values within rows with the same superscript letter were significantly different:

^a-b-c-e-d-f-g-h ^*P *< 0.05

**Figure 2:**
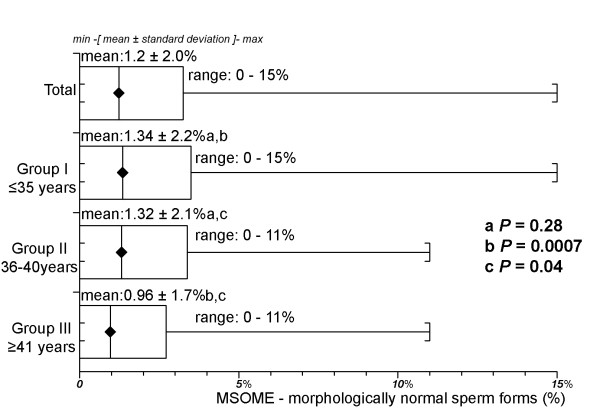
**Percentage of morphologically normal sperm forms by MSOME according to age for the three age groups**. There was no difference in the percentages of normal sperm in the two younger (I and II) groups (*P *= 0.28, Mann-Whitney U test). The percentage of normal sperm in the older group (III) was significantly lower than those in the younger (I and II) groups (*P *= 0.0007 and *P *= 0.04, Mann-Whitney U test).

The overall percentage of sperm with normal form, as analysed by MSOME, was 1.2 ± 2.0% (range 0-15%). The mean percentage of sperm with a normal form was 1.34 ± 2.2% (range 0-15%) in Group I, 1.32 ± 2.1% (range 0-11%) in Group II, and 0.96 ± 1.7% (range 0-11%) in Group III. There was no difference in the percentage of normal sperm in the two younger (I and II) groups (*P *= 0.28, Mann-Whitney U test). The percentage of normal sperm in the older group (III) was significantly lower than in either of the younger (I and II) groups (*P *= 0.0007 and *P *= 0.04, respectively, Mann-Whitney U test). Figure 2 summarises these results.

**Figure 3:**
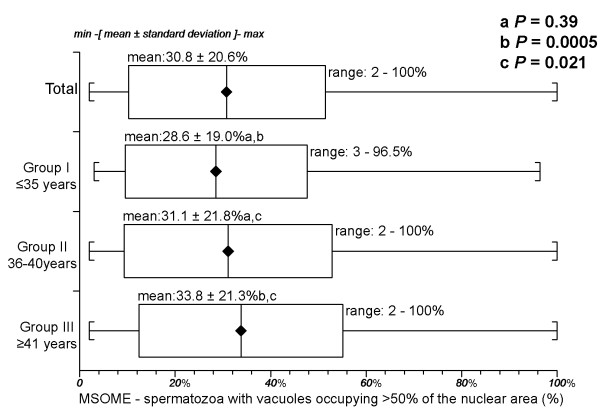
**Percentage of spermatozoa with large nuclear vacuoles (presence of one or more vacuoles occupying > 50% of the nuclear area) by MSOME according to age for the three age groups**. There was no difference in the percentages of spermatozoa with large nuclear vacuoles in the younger (I and II) groups (*P *= 0.39, Mann-Whitney U test). The percentage of spermatozoa with large nuclear vacuoles in the older group (III) was significantly lower than those in the younger (I and II) groups (*P *= 0.0005 and *P *= 0.021, Mann-Whitney U test).

The overall percentage of LNV spermatozoa was 30.8 ± 20.6% (range 2-100%). The mean percentages of LNV spermatozoa were 28.6 ± 19.0% (range 3-96.5%) in Group I, 31.1 ± 21.8% (range 2-100%) in Group II, and 33.8 ± 21.3% (range 2-100%) in Group III. There was no difference in the percentages of spermatozoa with large nuclear vacuoles between the younger (I and II) groups (*P *= 0.39, Mann-Whitney U test). The percentage of spermatozoa with large nuclear vacuoles in the older group (III) was significantly lower than those in both of the younger (I and II) groups (*P *= 0.0005 and *P *= 0.021, respectively, Mann-Whitney U test). Figure 3 summarises these results.

**Figure 4:**
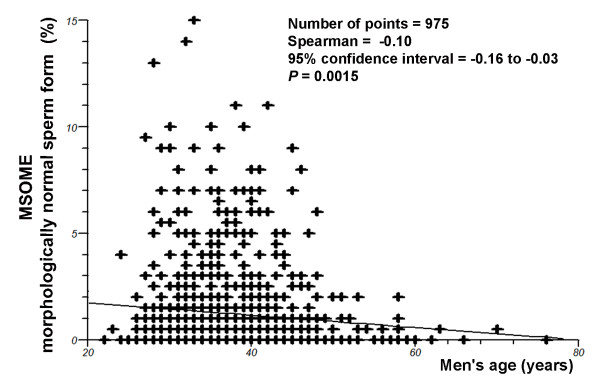
**Relationship between male age (years) and the percentage of morphologically normal sperm, as evaluated by MSOME**. Individual data points and a regression line are shown. Spearman rank correlation r = -0.1; *P *= 0.0015.

**Figure 5:**
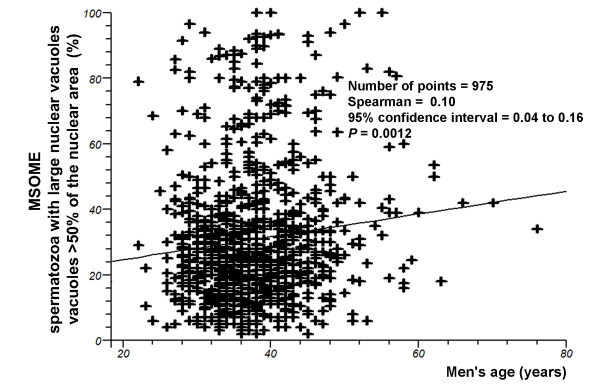
**Relationship between male age (years) and the percentage of spermatozoa with large nuclear vacuoles (presence of one or more vacuoles occupying > 50% of the nuclear area), as evaluated by MSOME**. Individual data points and a regression line are shown. Spearman rank correlation r = 0.10; *P *= 0.0012.

Regression analysis demonstrated a significant decrease in the incidence of normally formed sperm with increasing male age (*P *= 0.0015; Spearman's rank correlation r = -0.10) (Figure 4). However, there was a significant positive correlation between the percentage of spermatozoa with large nuclear vacuoles and male age (*P *= 0.0012, Spearman rank correlation r = 0.10) (Figure 5).

## Discussion

Our results demonstrate that the percentage of normal spermatozoa decreases significantly (*P *= 0.0015) and that the percentage of LNV spermatozoa increases significantly (*P *= 0.0012) with subject age in a large clinical sample of men undergoing infertility treatment or evaluation. Unfortunately, MSOME is not typically applied beyond sperm selection. In fact, to the best of our knowledge, only Braga *et al. *[10] have analysed the relationship between sperm morphology evaluated by MSOME and patient age. In contrast to the present results, those authors found no correlation between the frequency of morphologically normal spermatozoa as defined by MSOME and male age (*P *= 0.715). However, similar to the results of this study, a positive correlation was found between male age and the presence of nuclear vacuoles (large vacuoles *P *< 0.001; small vacuoles *P *< 0.001). It should be stressed that those authors defined the MSOME criteria for the morphologic normalcy of the sperm nucleus according to Cassuto *et al. *[29], while we used the criteria proposed by Bartoov *et al. *[20]. This difference may explain the conflicting results.

Although the correlation between age and sperm morphology by MSOME was significant, it could be considered weak (Spearman's r = -0.10 and r = 0.10). However, the correlation was similar to those found by authors using different sperm classification criteria, e.g., Brahem *et al. *[39] r = 0.026, not significant; Andolz *et al. *[2] r^2 ^= 0.020, *P *< 0.001; and Braga *et al. *[10] r^2 ^= 0.118 P *<*0.001. It is likely that other factors may influence the correlation between age and morphology. Unfortunately, few studies have used this type of statistical analysis, which makes the interpretation of these correlation values challenging.

In the present analysis, significant changes in sperm morphology were observed in most men 41 years old or older. Studies using other morphological sperm evaluation criteria have shown similar results. Mladenovic *et al. *[11] conducted a prospective study of 77 semen specimens and reported that abnormal spermatozoa were found mostly in patients over 40 years of age. Jung *et al. *[6], after adjusting for duration of sexual abstinence, observed that the percentage of morphologically normal spermatozoa was significantly lower (*P *< 0.01) in older men (n = 66; ≥ 50 years) than in younger men (n = 134; 21-25 years). Girsh *et al. *[7] examined a population of 484 men and showed that sperm morphology does not begin to diminish until age 40. Zhu *et al. *[9] analysed 998 subjects and showed that the percentage of normal sperm began to decrease slowly at age 30. However, differences in the study populations, age group cut-off points, and analysis methods prevent direct comparisons with this study's findings.

Our findings contrast with several studies that found no relationship between sperm morphology and age [39-41]. However, as noted in a review by Kidds *et al. *[41], the variation in the criteria used to analyse sperm morphology in each of these studies can explain this divergence. Some reports have associated an increase in the incidence of certain morphological abnormalities with age. Schwartz *et al. *[1] highlighted an increase in the percentage of microcephalic sperm and sperm with tail abnormalities with increasing age. Bujan *et al. *[12] observed that age is positively correlated with the percentage of microcephalic, macrocephalic, duplicate head-tailed and coiled tailed-spermatozoa and negatively correlated with the percentage of tailless spermatozoa. Centola *et al. *[4] demonstrated that the percentage of spermatozoa with tail defects and tapered heads showed a significant positive correlation with age (i.e., defects increased as age increased). Thus, differences in criteria are especially important because the count of specific abnormalities may differ depending on the classification used. Kidds *et al. *[41] cites the example that the WHO criteria [36] include more tail abnormalities than do the David criteria [12] and generally include different head abnormalities. On the other hand, MSOME places particular importance on fine sperm nuclear morphology. Nevertheless, our data are consistent with those of several other studies that used criteria other than MSOME [1-3,7,9,11].

The choice to analyse the percentage of LNV sperm in this study was motivated by the clinical implications of this phenotype. One plausible explanation for the increased frequency of spontaneous abortions, autosomal dominant disorders, aneuploidies, and other diseases is that older men may produce more spermatozoa with damaged DNA [42]. In fact, chromatin damage has been associated with male infertility and problems with conception and sustained pregnancy [43-47]. Furthermore, there is growing evidence associating sperm DNA damage with the risk of developmental abnormalities [17,18]. Bartoov *et al. *[20] and Berkovitz *et al. *[34], based on electron microscopy data, assumed that nuclear vacuoles indicate abnormal chromatin. Other studies confirmed the association between nuclear vacuoles at high magnification and chromatin damage. Berkovitz *et al. *[30] graded the severity of nuclear morphological defects, principally highlighting the presence of large vacuoles and suggesting that the vacuolisation of the sperm nucleus reflects some underlying chromosomal or DNA defect. Garolla et al. [25] showed that the presence of nuclear vacuoles affects mitochondrial function, chromatin status, and aneuploidy rate. Franco at al. demonstrated an association between large nuclear vacuoles and both DNA fragmentation and denaturation in the spermatozoa [24,27] and an association between large nuclear vacuoles and abnormal chromatin packaging [24,27]. Moreover, Oliveira et al. [23] and Wilding et al. [28] associated the presence and extent of nuclear vacuoles with DNA damage. As reported by other authors, the present study also observed an increase in sperm DNA fragmentation that was directly related to increased patient age. Therefore, these data indirectly confirm the previously described correlation between the presence of LNV and DNA damage.

The accuracy with which the morphological normality of spermatozoa can be assessed depends on the resolving power of the optical magnification system. Spermatozoa that appear morphologically normal at 1000× magnification may in fact carry various structural abnormalities that can only be detected at higher magnifications (> 6000×). The improvement in observation is mainly due to the replacement of Hoffman modulation contrast with the Nomarski interferential modulation contrast. Increases in the resolution of the optical system were made possible by the development of optical techniques such as Hoffman differential interference, which allowed for the visualisation of morphological characteristics of oocytes and spermatozoa. However, small cells such as sperm may have some morphological abnormalities (e.g. anomalies on the intermediate piece and the presence of vacuoles), which are not easily observed at the resolution offered by Hoffman 40x objectives. To allow for a more detailed morphological analysis of small cells, higher optical resolutions provided by the 100× DIC objectives are more appropriate [28]. Thus, the use of MSOME may represent a potential improvement in the morphological analysis of the sperm. The resolving power offered by MSOME enables the identification of spermatozoa with intranuclear vacuoles that would not be detected with more conventional evaluation methods. For example, Bar-Chama *et al. *[48], employing the Tygerberg criteria, analysed the number of sperm vacuoles in a series of 1295 fresh post-processed sperm samples. They found vacuolated nuclei in only 19.5% (253) of the total analysed sperm; 80.5% (1042) had no vacuoles. On the other hand, MSOME revealed averages from 30-40% [30] to > 90% [23] of spermatozoa with vacuolated nuclei.

Bartoov *et al. *[20] emphasised that, while routine morphological examination is applied to semen samples as a whole, MSOME concentrates only on the motile fraction of spermatozoa. Because some morphological defects, such as large vacuoles, can be revealed during sperm movement, motility provides an advantage for morphological observation [34]. Furthermore, the analysis of only motile spermatozoa by MSOME has an additional advantage in that it will provide information on the sample fraction with greater real fertilisation and development potential. Although other analytic criteria can employ high magnification observation, the procedures used (fixation and staining) do not allow the selective analysis of the motile portion alone.

## Conclusions

In summary, the results of this study clearly demonstrate a consistent decline in semen quality, in terms of morphology judged by MSOME (i.e., a decrease in the percentage of normal spermatozoa and a concomitant increase in the percentage of LNV spermatozoa), with an increase in patient age in an infertile population. Considering the relationship between nuclear vacuoles and DNA damage, these age-related changes suggest that advanced paternal age may be associated with an increased risk of unsuccessful and abnormal pregnancy as a consequence of fertilisation with damaged spermatozoa. This information may be useful in the clinical management of male infertility. Based on the clinical/laboratory findings on the repercussions of possible DNA damage in offspring [49] and given that sperm nuclear vacuoles can be evaluated more precisely at high magnification by MSOME [20], the present results support the routine use of MSOME for ICSI and as a criterion for semen analysis with potential clinical repercussions.

## Competing interests

The authors declare that they have no competing interests.

## Authors' contributions

LFIS designed and coordinated the study. All authors were responsible for the data collection, analysis, and interpretation presented in the manuscript. LFIS, JBAO, CGP and JGF performed the statistical analyses and wrote the manuscript; JGF reviewed the manuscript. All authors read and approved the final manuscript.
